# MicroEXPERT: Microbiome profiling platform with cross‐study metagenome‐wide association analysis functionality

**DOI:** 10.1002/imt2.131

**Published:** 2023-08-17

**Authors:** Pengshuo Yang, Jialiang Yang, Haixia Long, Kaimei Huang, Lei Ji, Hanyang Lin, Xiuli Jiang, Arthur Kairui Wang, Geng Tian, Kang Ning

**Affiliations:** ^1^ Key Laboratory of Molecular Biophysics of the Ministry of Education, Hubei Key Laboratory of Bioinformatics and Molecular‐imaging, Center of AI Biology, Department of Bioinformatics and Systems Biology College of Life Science and Technology, Huazhong University of Science and Technology Wuhan Hubei China; ^2^ Institute of Medical Genomics Biomedical Sciences College, Shandong First Medical University Jinan Shandong China; ^3^ Department of Sciences Geneis Beijing Co., Ltd. Beijing China; ^4^ Department of Sciences Qingdao Geneis Institute of Big Data Mining and Precision Medicine Qingdao China; ^5^ Department of Sciences Academician Workstation, Changsha Medical University Changsha China; ^6^ Department of Information Science Technology Hainan Normal University Haikou China; ^7^ Department of Mathematics Zhejiang Normal University Jinhua China; ^8^ Department of Sciences Sequenxe Biological Technology Co., Ltd. Xiamen China; ^9^ Department of Biology Dulwich College London UK

## Abstract

The framework of the MicroEXPERT platform. Our Platform was composed of five modules. Data management module: Users upload raw data and metadata to the system using a guided workflow. Data processing module: Uploaded data is processed to generate taxonomical distribution and functional composition results. Metagenome‐wide association studies module (MWAS): Various methods, including biomarker analysis, PCA, co‐occurrence networks, and sample classification, are employed using metadata. Data search module: Users can query nucleotide sequences to retrieve information in the MicroEXPERT database. Data visualization module: Visualization tools are used to illustrate the metagenome analysis results.

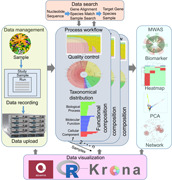

## INTRODUCTION

The field of metagenomics has emerged as a potent tool for studying microbial communities in different environments (known as biomes) [1]. Large‐scale microbiome research, such as the Human Microbiome Project [2, 3], the *Tara* oceans project [4, 5, 6], and the earth microbiome project (EMP) [7], have emerged to investigate microbial communities, and this is largely due to the rapid development of sequencing technology [8, 9, 10]. These metagenome projects have generated voluminous amounts of data and urged microbiome research to become a data‐driven science [11, 12, 13].

Among the factors contributing to the success of these projects, of particular note are the successful combinations of environmental factors (i.e., metadata) to investigate the inherent distribution and biological significance in microbial communities [14, 15, 16]. By combining the metadata with the metagenome, our understanding of the microbial communities has improved significantly [17, 18, 19]. Metagenome‐wide association studies (MWAS) that apply the concept of a genome‐wide association study for the association analysis can provide a high‐resolution investigation of associations between the microbiome and their environmental parameters [20, 21, 22]. MWAS would yield a collection of statistical connections or models between microbial communities and various health or environmental situations [23, 24]. These associations include the environmental factors that shape the microbial communities [25, 26] as well as the identification of microbial functions that are enriched in specific environments [27, 28, 29]. The MWAS analysis can evaluate the potential effects of clinical and lifestyle factors on the microbiome that is related to human health [29, 30, 31]. Hence, many databases have been constructed to maintain metagenome data and metadata for mining meaningful interpretations based on metagenomes from different environments (i.e., biomes) [32, 33, 34], such as MGnify [35], the Integrated Microbial Genomes (IMG) systems [36, 37] and the Sequence Read Archive (SRA) database [36, 38]. However, faced with a huge amount of metagenome data and the corresponding heterogeneous metadata, a platform is required to utilize the metadata to decipher the relationship between biomes and metagenomes.

In this work, we introduce the MicroEXPERT platform, a sophisticated system engineered to proficiently manage, analyze, and mine metagenomic data with precision and depth. First, an efficient and state‐of‐the‐art database management system was designed to support long‐term data preservation. Second, we designed a search module to facilitate multilevel exploration (from gene to biome) of the metagenome. Finally, the MWAS module were designed with various statistical techniques, an intuitive graphical user interface, and an interactive analysis result. We are dedicated to constructing an accessible, powerful, and informative MWAS module of microbial communities.

## RESULTS

### The interface of the MicroEXPERT platform

**Figure 1:**
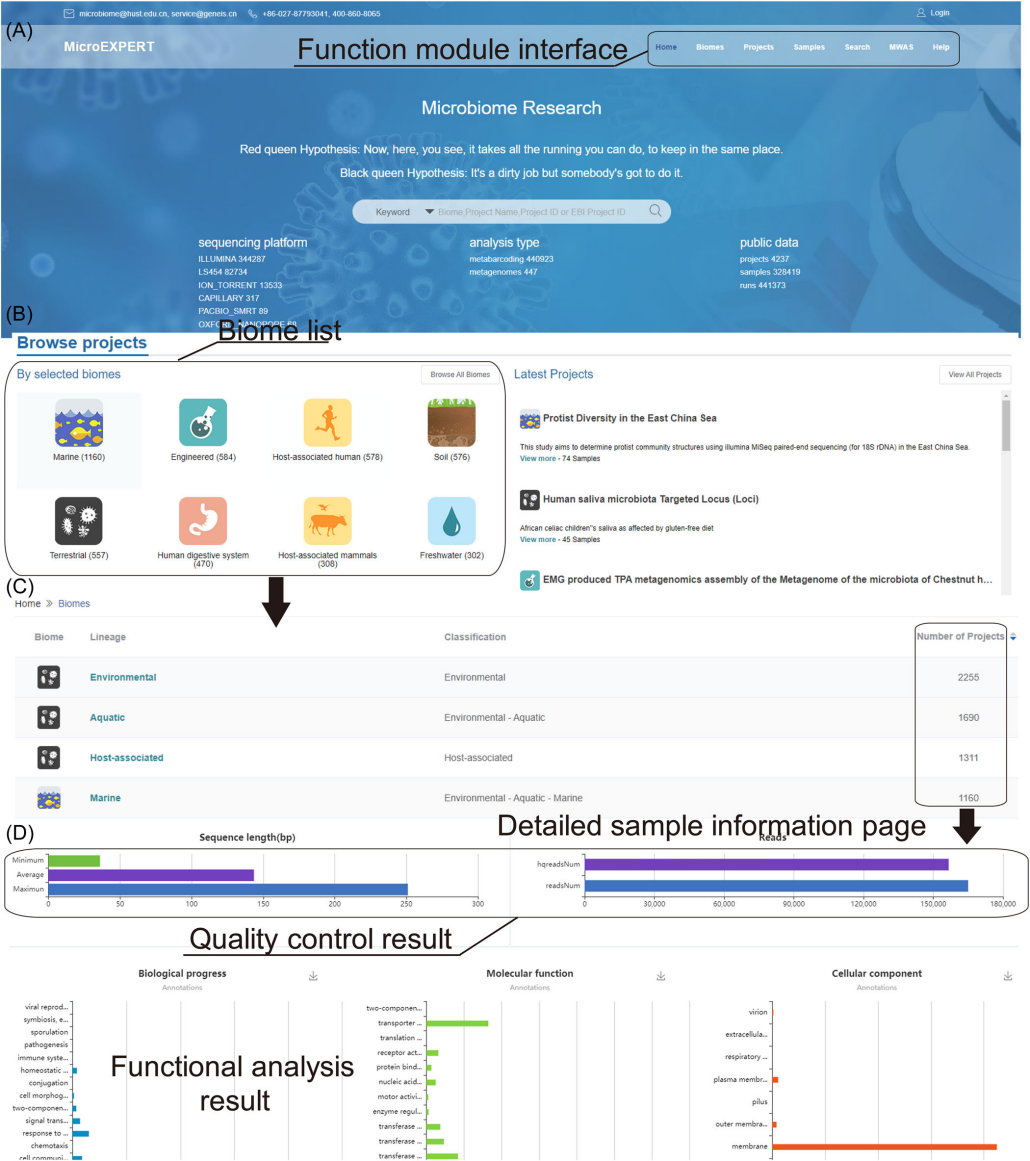
Screenshots and examples of user cases in MicroEXPERT (https://microexpert.aimicrobiome.cn/). (A) Homepage of the MicroEXPERT. Interfaces to other modules are supplied, including the introduction and the statistical information of MicroEXPERT. (B) Projects classified by biome information. The home page exhibits the biomes that hold the most projects. The latest submitted projects are also shown. (C) A screenshot of the biome information. All of the samples are classified into 235 biomes, each of which links to the project's details. (D) The example of sample information page. For each sample, the quality control result of the upload sample was illustrated. Low‐quality sequences were filtered out by steps. Moreover, the functional results were divided into three categories: biological process, Cellular Composition, and Molecular functions.

The interface of MicroEXPERT is designed to be user‐friendly and full‐featured. The home page of MicroEXPERT exhibits the database introduction and provides interfaces for all the analyses at the top of the page (Figure 1A). MicroEXPERThas retrieved 4.5 TB of raw data and related data. The “Biome” page depicts samples that are classified into 235 biomes referred to by MGnify [35] (Figure 1B,C). The “Project” portal stores the comprehensive process result on the samples webpage (Figure 1D). To facilitate search and navigation, the “Samples” webpage depicts the referred samples and run information (Figure 1D). With a query nucleotide sequence, the “Search” webpage provides a comprehensive set of bioinformatics tools for gene annotation, species identification, and sample source monitoring. The “MWAS” provides more than 40 tools for performing in‐depth data mining based on our database's stored metadata and sample analysis results. The “Help” page illustrates the platform introduction and data processing pipeline.

### Data management of the MicroEXPERT platform

The MicroEXPERT platform offers a data archival service designed for storing both the raw reads and the processed data results. In addition, our comprehensive database facilitates seamless data submission, integrated with a sophisticated data management system (https://microexpert.aimicrobiome.cn/welcome). This intuitive interface assists users in selecting the project name, uploading samples and runs, and categorizing the samples manually by providing metadata in a logical, step‐by‐step manner. To maintain data integrity, our diligent backstage staff performs manual checks on the uploaded sample information. If the sample information is found to be incomplete or the sample quality fails to meet the required standards, the user is promptly notified via email.

### MWAS in MicroEXPERT

**Figure 2:**
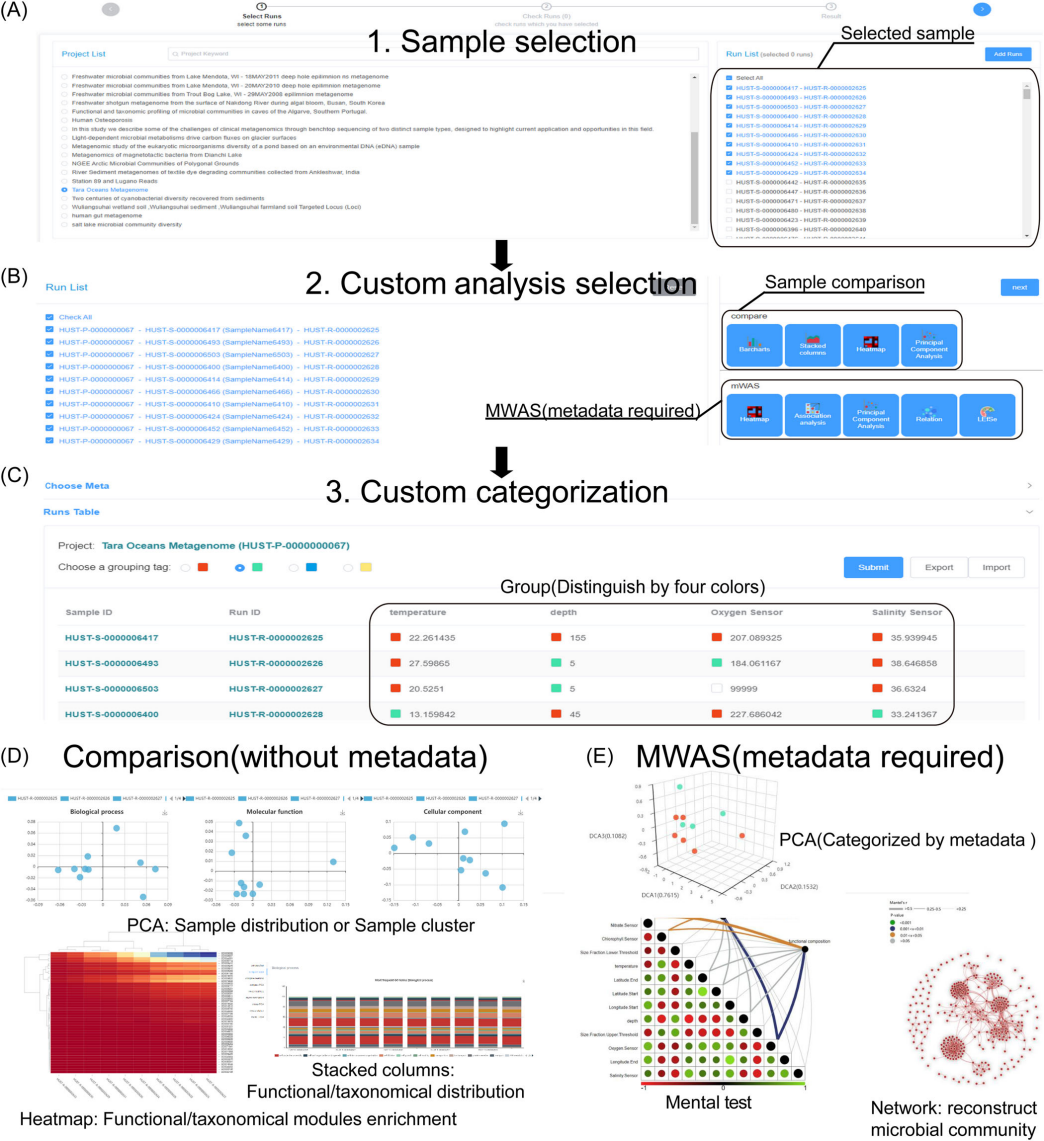
A screenshot of the MWAS workflow (https://microexpert.aimicrobiome.cn/mwas). (A) Sample selection. Users can choose all the samples processed by the analysis workflow in the MicroEXPERT database. Cross‐study selection is applicable. (B) Custom analysis selection. In this step, the users can check the selected samples and choose the analysis tools (MWAS analysis and comparison methods). At least 10 samples are recommended for MWAS analysis. (C) Metadata categorization. For the selected samples, their metadata in our database are uploaded. The users can categorize these samples into at most four groups labeled with the chosen color. Alternatively, users can upload the group information by importing the table from your local computer. (D) Sample comparison. The sample is compared using different statistical tools such as a principal component analysis (PCA) analysis, a heatmap, and stacked columns. (E) MWAS analysis. Based on the categorization information and taxonomical or functional composition, the association between the metagenome and metadata is analyzed using variety of statistical tools such as heatmap, mental test, PCA, and network.

The MicroEXPERT platform is dedicated to providing a comprehensive set of well‐established statistical methods for MWAS. For instance, the MWAS analysis within MicroEXPERT offers a data set comprising 10 samples from the Tara Oceans data set, collected at two distinct temperatures (Figure 2A). By classifying the samples based on temperature, the MWAS analysis can identify the differences between the two groups by examining the influence of environmental factors through environmental association analysis, investigating the association patterns within the bacterial community using co‐occurrence network analysis, and identifying key species responsible for the observed differences through LefSE analysis. Furthermore, the platform allows for the construction of a machine learning model, specifically the Random Forest function, which can be employed to classify samples based on their temperature variations.

First, to initiate the MWAS analysis, the user is required to select the project of interest along with the corresponding runs (Figure 2A). Upon clicking the “mWAS” link on the homepage, users will be directed to the project selection page. Once the project and run information has been selected, the subsequent steps involve addressing batch effects and normalization (DESeq. 2, https://rdrr.io/bioc/DESeq.2/man).

Second, based on the selected runs, the statistical methods should be specified (Figure 2B). We supply a variety of statistical tools for the run comparison (independent of the metadata) and the MWAS analysis (dependent on the metadata). The functions encompass key components of metagenome data analysis, comprising data visualization (Heatmap), Dimensionality Reduction (PCA), Correlation calculation (Environmental association analysis and Co‐occurrence network), biomarker detection (LEfSe), and machine learning modeling (Random Forest).

Third, once the selection of statistical methods is completed, the metadata should be uploaded (Figure 2C). Users can choose different color labels to group the data and divide them into up to four distinct groups. For batch grouping, we offer a service to download the metadata in a tab‐separated file format (“.tsv”) and reupload the categorized metadata after editing on their local computer.

Finally, all the run comparison results and MWAS analysis results are illustrated in an app list (Figure 2D). Similarly, the MWAS result can also illustrate the metadata and runs in multiple angles and dimensions (Figure 2E): The PCA analysis directly measures the sample distribution among the different categorizations. Then the heatmap and mental test result measure the driven taxonomical models and environmental factors, respectively. The biomarker then further detects the differences on the species level and exhibits their potential relationships by co‐occurrence network. Finally, our platform performs a machine‐learning model based on a random forest algorithm.

### Search engine

**Figure 3:**
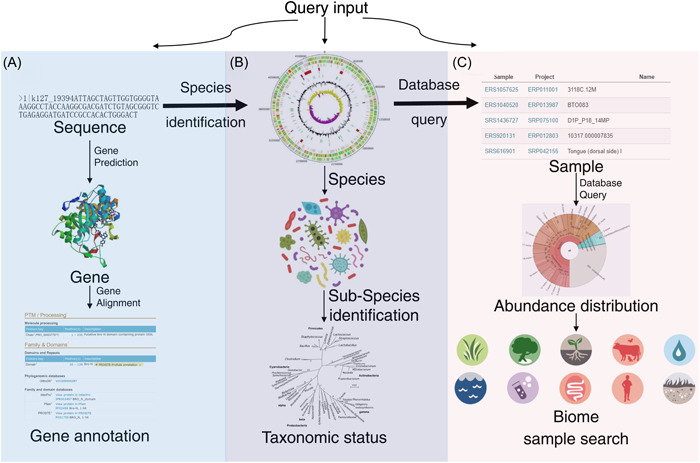
Investigation the gene‐species‐sample relationship with one sequence (https://microexpert.aimicrobiome.cn/search). (A) Gene annotation workflow. For the query nucleotide sequences, the MicroEXPERT platform searches the genes in the NCBI nucleotide database using the tool BLAST+ (version 2.5.1). The detailed matched information based on the NCBI nucleotide database are also illustrated. (B) Species identification workflow. To identify the possible source species of the query sequence, Kraken2 (version 2.0.7) and MetaPhlAn4 (version 4.0.2) are used. The pedigree‐based and phylogenetic relationship of the matched species is deduced using the NCBI taxonomy database. (C) Sample search workflow. To explore the distribution of samples and the biome of the query species in the database, all analysis results and sample information are stored in a separate table to return the abundance information of the query species in different environments and samples. The samples containing the queried species are illustrated according to their relative abundance in the related samples.

Many metagenome databases excel at data querying, visualization, and comparative analysis of processed data. However, to enhance the data search capabilities and streamline operations further, it is necessary to develop an advanced data search mechanism that leverages the taxonomical distribution of the metagenome (Figure 3). In this regard, we have devised a multilevel data mining pipeline that establishes connections between gene annotation, species identification, sample mapping, and a query sequence. Initially, at the gene level, gene annotation functionality (Figure 3A) is implemented using BLAST+ (version 2.7.1) and the NR database (retrieved on 2018.1.10). At the species level (Figure 3B), the gene annotation includes a species record for the matched gene, utilizing the source species information from the NR database. To identify the accurate species, we employ Kraken [39], a fast and precise species identification tool. On the biome level (Figure 3C), all taxonomical analysis results are stored in a single MySQL database, enabling the exploration of species distribution within the database samples.

Using the example sequence in the Blast function (https://microexpert.aimicrobiome.cn/search): This 560 bp sequence has been identified as the gene of *Alteromonas macleodii ATCC 27126* (gene ID: CP003841) using the Blast tool. This genus predominates in the *Tara* Oceans Project, which extensively sequenced the ocean microbiome (highest relative abundance: 5.69%). This particular sample was collected from the surface at a depth of 5 meters, aligning with the known physiology and ecological preferences of the *Alteromonas* genus [4].

### Comparison with other platforms

Numerous noteworthy platforms have been developed to facilitate microbiome analysis (Table 1). However, many of these tools primarily focus on raw data management and processing, offering limited support for advanced statistical analysis and data mining functions (Table 1). In contrast, MicroEXPERT serves as a valuable complement to existing data repositories and search capabilities. Through a meticulous comparison of these web‐based tools (Table 1), it becomes apparent that MicroEXPERT distinguishes itself by offering a unique array of features and functions.

**Table 1:** Comparison of MicroEXPERT with other web‐based tools. The URL for each tool is given below the table.

Platform	Data management			Data analysis			Metagenome‐wide association study			
	User registration	Raw data upload	Data search^a^	Raw data process	Result visualization^b^	Mining Tools^c^	Online categorization	Online visualization	Result download	Code avaliable
MicroEXPERT	Yes	Yes	Yes	Yes	Yes	Yes	Yes	Yes	Yes	Yes
MicrobiomeAnalyst2	No	No	No	Yes	No	No	No	No	Yes	No
Qiita	Yes	Yes	No	Yes	No	No	Yes	Yes	Yes	No
Mgnify	Yes	Yes	Yes	Yes	Yes	Yes	No	No	No	No
CoMet2	Yes	No	No	Yes	Yes	No	No	No	Yes	No

MicrobiomeAnalyst2: www.microbiomeanalyst.ca.

Qiita: qiita.ucsd.edu.

Mgnify: www.ebi.ac.uk/metagenomics.

CoMet2: comet2.gobics.de.

^a^ Data search means the tools or database for sequences search.

^b^ Result Visualization means the analysis result could be online visible.

^c^ Mining tools means the online server for sequence searching, species identification, and so on.

### Implementation and utility of the tools and workflows

We adopted the architecture of separating the front and back end, meaning that the data are stored in the background and the real‐time analysis is implemented on another server. This was done to reduce the front and back‐end communication costs and improve development efficiency. The web interface was developed using Hypertext Preprocessor (PHP). The database was constructed based on Mangodb for the project‐associated information, metadata, and analysis result. The tools and workflows were installed using Docker. MicroEXPERT is powered by two computational servers: The first server, dedicated to data management, has a CPU with 16 cores, 32GB of memory, and a storage capacity of 16TB. The second server, utilized for online MWAS analysis, features a CPU with 32 cores, 256GB of memory, and a storage capacity of 4TB.

## DISCUSSION

The bottleneck in online MWAS analysis lies in the autonomous recognition of metadata, which possesses high‐dimensional and heterogeneous characteristics. To tackle this problem, several measures have been implemented to enhance the system's accuracy and robustness: (1) Project Selection Step: The MWAS analysis is restricted to samples within the same project. Samples from different projects are not eligible for analysis. (2) Sample Categorization: We have developed a dedicated webpage where users can categorize their samples based on up to four different criteria, facilitating more refined analysis. (3) Preprocessing Script Customization: After conducting a series of systematic tests, we have customized the preprocessing script for each MWAS method, ensuring compatibility with various data types and values. (4) Minimum Number of Runs: Considering the importance of statistical significance in MWAS results, we recommend a minimum of 10 runs for analysis on our website. This criterion helps guarantee reliable and meaningful MWAS outcomes.

To the best of our knowledge, MicroEXPERT is the first metagenome database that focuses on online data mining of microbiome that has been preprocessed by MicroEXPERT database and tailored big data analytic tools. MicroEXPERT will be updated every 6 months to incorporate the most recent developments in metagenome research and MWAS analysis. We think that ongoing work in this integrated database and analytical platform will contribute to the worldwide mining of metagenome data and human health.

## CONCLUSION

As a platform for the collection and analysis of microbial samples, MicroEXPERT retrieved 4236 projects, covering 328,417 samples with detailed taxonomical and functional profiles. Our server has collected numerous projects to support our MWAS analysis and gene search module. What is worthy of our continued efforts is that more data should be collected for deeper insights into the metagenome. Moreover, improving the effectiveness of MWAS will require overcoming challenges in microbiome sampling, sequencing, bioinformatics analysis, and functional characterization. Consequently, in future updates of MicroEXPERT, we will develop an analyzing pipeline with higher accuracy and resolution for identifying microbial species and functions. Additionally, more advanced artificial intelligence techniques, such as deep learning for sample classification and host status predictions, as well as improved data mining techniques, such as gene mining tools, and multiomics data integration tools may be implemented.

## AUTHOR CONTRIBUTIONS

Kang Ning, Geng Tian, Pengshuo Yang, and Jialiang Yang conceived and proposed the idea, and designed the study. Pengshuo Yang, Haixia Long, and Kaimei Huang performed the experiments. Lei Ji, Kang Ning, Hanyang Lin, Kaimei Huang, Geng Tian, and Kang Ning analyzed the data. Kang Ning, Pengshuo Yang, Xiuli Jiang, Arthur Kairui Wang, Jialiang Yang, and Kang Ning contributed to the editing and proofreading of the manuscript. All authors read and approved the final manuscript.

## CONFLICTS OF INTEREST STATEMENT

Jialiang Yang, Lei Ji, and Geng Tian are employed by Geneis Beijing Co., Ltd. Haixia Long and Kaimei Huang are employed by Sequenxe Biological Technology Co., Ltd. The remaining authors declare no conflict of interest.

## Data Availability

The MicroEXPERT platform is available at https://MicroEXPERT.aimicrobiome.cn/. Supplementary materials (scripts, graphical abstract, slides, videos, Chinese translated version, and update materials) may be found in the online DOI or iMeta Science http://www.imeta.science/.
